# Socioeconomic Gradients in Prostate Cancer Incidence Among Canadian Males: A Trend Analysis From 1992 to 2010

**DOI:** 10.1177/10732748211055272

**Published:** 2021-12-10

**Authors:** Mohammad Hajizadeh, Ashley Whelan, Grace M. Johnston, Robin Urquhart

**Affiliations:** 1 School of Health Administration, Faculty of Health, 3688 Dalhousie University, Halifax, NS, Canada; 2 12361 Faculty of Medicine, Dalhousie University, Halifax, NS, Canada; 3 Cancer Care Program, and School of Health Administration, 3688 Dalhousie University, Halifax, NS, Canada; 4 Department of Community Health and Epidemiology, Faculty of Medicine, 3688 Dalhousie University, Halifax, NS, Canada

**Keywords:** prostate cancer incidence, income, education, inequalities, Canada

## Abstract

**Introduction:**

Understanding the effects of socioeconomic status on cancer incidence and their trends over time will help inform public health interventions for cancer control. This study sought to investigate trends in socioeconomic inequalities in prostate cancer incidence among Canadian males.

**Methods:**

Using a census division level dataset (n = 280) constructed from the Canadian Cancer Registry, Canadian Census of Population (1992, 1996, 2001, 2006) and 2011 National Household Survey, we examined the effect of socioeconomic status on prostate cancer incidence among Canadian males between 1992 and 2010. The age-adjusted concentration index was used to quantify education/income-related inequalities in prostate cancer incidence.

**Results:**

The crude prostate cancer incidence increased from 115 to 137 per 100 000 males in Canada from 1992 to 2010 with a peak in 2007. The rate increased significantly in all but three of four western provinces. The age-adjusted concentration index showed a higher concentration of prostate cancer diagnoses among males living in high-income neighbourhoods in Canada in particular from 1996 to 2005. In contrast, the index was higher among males living in less-educated neighbourhoods in the most recent study years (2006–2010).

**Conclusions:**

The concentration of new prostate cancer cases among high-income populations in Canada may be explained by the rise of opportunistic screening of asymptomatic males; however, this should be studied in further detail. Since we found a higher incidence rate of prostate cancer among less-educated males in Canada in recent years, risk-benefit investigation of primary prevention and opportunistic screening for less-educated males is advised.

## Introduction

As the second leading cause of death worldwide, cancer is an important subject of public health research.^1^ It is estimated that one in two Canadians will develop cancer throughout their life, and approximately one in four Canadians will ultimately die from cancer.^2,3^ Prostate cancer (PCa) is the second most prevalent cancer in males, and the sixth leading cause of death in males globally.^4^ It is especially prevalent amongst developed countries: in 2020, it accounted for 21% of all new cancer cases and 10% of all cancer-related deaths among Canadian males.^5^

PCa is an adenocarcinoma of the prostate gland and can remain indolent and asymptomatic for a long period of time. As a result, the rapid uptake of opportunistic prostate-specific antigen (PSA) screening in the early 1990s led to a substantial increase in the incidence rate of PCa in more developed countries and among higher socioeconomic status (SES) populations.^6^ Concurrently, randomized control trials of PSA PCa screening were being carried out in the United States and Europe.^7,8^ The results suggested that while the PSA PCa screening reduced the mortality rate from prostate cancer by 20% after 5–7 years of follow-up in seven European countries, the screening did not decrease the rate of death from PCa after 9 years of follow-up. PSA screening remains controversial.^9^

SES is an important factor in health inequalities, where lower SES has been linked to poorer health outcomes spanning multiple domains including mental health, non-communicable diseases and cancer.^10-14^ Although age, race and family background are the main risk factors for PCa,^4,6,15-17^ the existing literature suggest that SES affects PCa incidence. Several of these studies from the United States cite possible connections to the delivery of health services in that country.^18-23^ SES is also associated with several environmental and lifestyle risk factors that may jointly impact the PCa burden.^20,24^ Dietary elements such as animal fat have been thought to be involved in the incidence of PCa due to the high proportion of alpha-linolenic acid to linoleic acid.^25^ Other nutritional sources have been shown by some studies to decrease the risk of PCa, such as high vegetable intake, cruciferous vegetables and soy products.^26,27^ Some studies have shown that males receiving fewer hours of sleep per night and certain night shift workers are at a higher risk of developing PCa.^28,29^ Psychosocial stressors indirectly affect one’s risk of developing PCa through biological and behavioural pathways.^30^ Higher levels of work-related stress were found to have a corresponding increased risk of developing PCa compared to their peers.^31^

Although there have been studies showing socioeconomic inequalities in PCa rates such as the relationship between PSA screening and incidence and mortality in Finland,^9^ mortality from a Danish cohort study^32^ and mortality in Taiwan,^33^ there is no study that investigates trends over time in socioeconomic inequalities in PCa incidence in Canada. This study aims to fill this gap in the literature by assessing income and education inequalities in PCa incidence in Canada over the period between 1992 and 2010. Obtaining a more complete understanding of socioeconomic inequalities of PCa incidence in Canada can inform policy decision-making and future public health initiatives to reduce PCa burden for Canadians.

## Methods

### Data and Variables

This study used a census division (CD) level dataset (n = 280) constructed from the Canadian Census of Population (CCP, 1991, 1996, 2001, 2006), 2011 National Household Survey (NHS) and Canadian Cancer Registry (CCR) to measure income and education inequalities in PCa incidence over the period between 1992–2010. This was the period in which the CCR data are available for all provinces. The CCR is a population-based cancer registry that collects tumour-specific data on diagnosed cancer cases.^34^ In order to identify males with PCa in the CCR data, the World Health Organization’s International Classification of Diseases for Oncology, third edition (ICD-O-3) code C61.9 was used.

As the CCR does not contain information on SES, the CCP and NHS were used to derive information on income and education required to measure socioeconomic inequalities in the incidence of PCa in Canada. We calculated the total number for males, mean and median equivalized annual household income and the proportion of individuals with a bachelor’s degree and higher for each CD using information available in the four CCPs and the 2011 NHS. The annual household incomes were equivalized using a square root scale (annual household income/square root of the household size)^35^ to take into account household size. As the CCR does not contain the CD of the males, we identified the CD of the males in the CCR using the Postal Code Conversion File Plus (PCCF+) Version D software.^36^ The demographic (number and age profile of males), income and education characteristics of each CD, calculated from the CCP/NHS data, was then linked to the number of PCa incident cases in each CD that were obtained from the CCR. CCP/NHS data were collected only in every fifth year; thus, we linked information derived from the 1992 CCP to 1992–1993 CCR, 1996 CCP to 1994–1998 CCR, 2001 CCP to 1999–2003 CCR, 2006 CCP to 2004–2008 CCR, and 2011 NHS to 2009–2010 CCR. We used the constructed linked dataset to measure the PCa incidence rate for each CD and quantify income and education inequalities in PCa incidence in Canada over the study period.

### Statistical Analyses

#### Measuring socioeconomic inequalities in PCa incidence

A summary measure of the concentration index ( C ) was used to quantify socioeconomic inequalities in PCa incidence. The C , which captures inequality across the entire spectrum of SES groups, is estimated based on the concentration curve ( CC ). The CC plots the cumulative proportion of the population ranked by ascending order of a socioeconomic variable (e.g. income or education) on its *x*-axis against the proportion of a health variable (PCa incidence) on its *y*-axis. If the CC overlaps with the 45-degree line, it indicates that health outcome is similar across all SES groups. The C is measured as twice the area between the 45-degree (perfect equality) line and the CC . If the CC lies below (above) the line of perfect equality, it implies that the health outcome (PCa incidence) is concentrated among the low (high) SES population.^37^ The C varies from −1 to 1. The zero value of C suggests perfect equality. The negative value of the C suggests that the health variable (PCa incidence) is more concentrated among low SES population and vice versa.

The ‘convenient regression’ formula to measure the C is as follows^38^

(1) $$2{\text{σ}}_{\text{FR}}^{2}\left(\frac{{\text{PCa}}_{\text{i}}}{\text{Mean}}\right)=\text{α}+{\text{γFR}}_{\text{i}}+{\text{ε}}_{\text{i}}.$$

where ${\text{PCa}}_{\text{i}}$ denotes PCa incidence for CD i , Mean is the average PCa incidence rate for all CDs, α is the intercept. ${\text{FR}}_{\text{i}}=\text{i}/\text{n}$ indicates the fractional rank in the SES distribution for CD i ( i=1  for the lowest SES CD and i=n for the highest SES CD). The ${\text{σ}}_{\text{FR}}^{2}$ is the variance of FR . The ordinary least squares estimate of γ represents the crude C .

The age-standardized (adjusted) socioeconomic inequality can be calculated using an indirectly standardized C by including the age-standardizing variables in the regression formula as follows^39^

(2) $$2{\text{σ}}_{\text{FR}}^{2}\left(\frac{{\text{PCa}}_{\text{i}}}{\text{Mean}}\right)=\text{α}+{\text{φFR}}_{\text{i}}+\overset{16}{\underset{\text{k}=1}{\sum }}{\text{β}}_{\text{k}}\text{A}\_{\text{G}}_{\text{ki}}+{\text{ν}}_{\text{i}},$$

where $\text{A}\_{\text{G}}_{\text{ki}}$ is the proportion of individuals in the age-group k (16 five year age-group variables with an open-ended 85+ age group, except for a reference group) for the CD i and ${\text{β}}_{\text{k}}$ is the related coefficients for $\text{A}\_{\text{G}}_{\text{k}}$ . The ordinary least squares estimate of φ and its standard error determines the magnitude and standard error of the age-standardized C . Total number of males in each CD was used as a weight in the calculation of the age-standardized C . The age-standardized C was measured using the three SES indicators: average and median household equivalized income, and the proportion of people with a bachelor’s degree or higher. A *P*-value of less than 0.05 was considered statistically significant.

#### Analysing trends in the incidence and socioeconomic inequalities

 Linear trend analyses were performed to examine changes in the crude PCa incidence and socioeconomic inequalities in the incidence of PCa over the period between 1992 and 2010. We regressed the crude PCa incidence rates or the age-standardized C s (depending on whether we assessed the trend in the incidence or socioeconomic inequalities) on time corresponding to the study years. A statistically significant positive trend coefficient for the crude PCa incidence rate implies an increasing trend in the incidence rate over time and vice versa. A negative value of the trend coefficient for age-standardized Cs suggests an increasing trend in the concentration of the PCa incidence among lower-income/education population over time and vice versa.

## Results

### Crude Prostate Cancer Incidence

**Figure 1.**
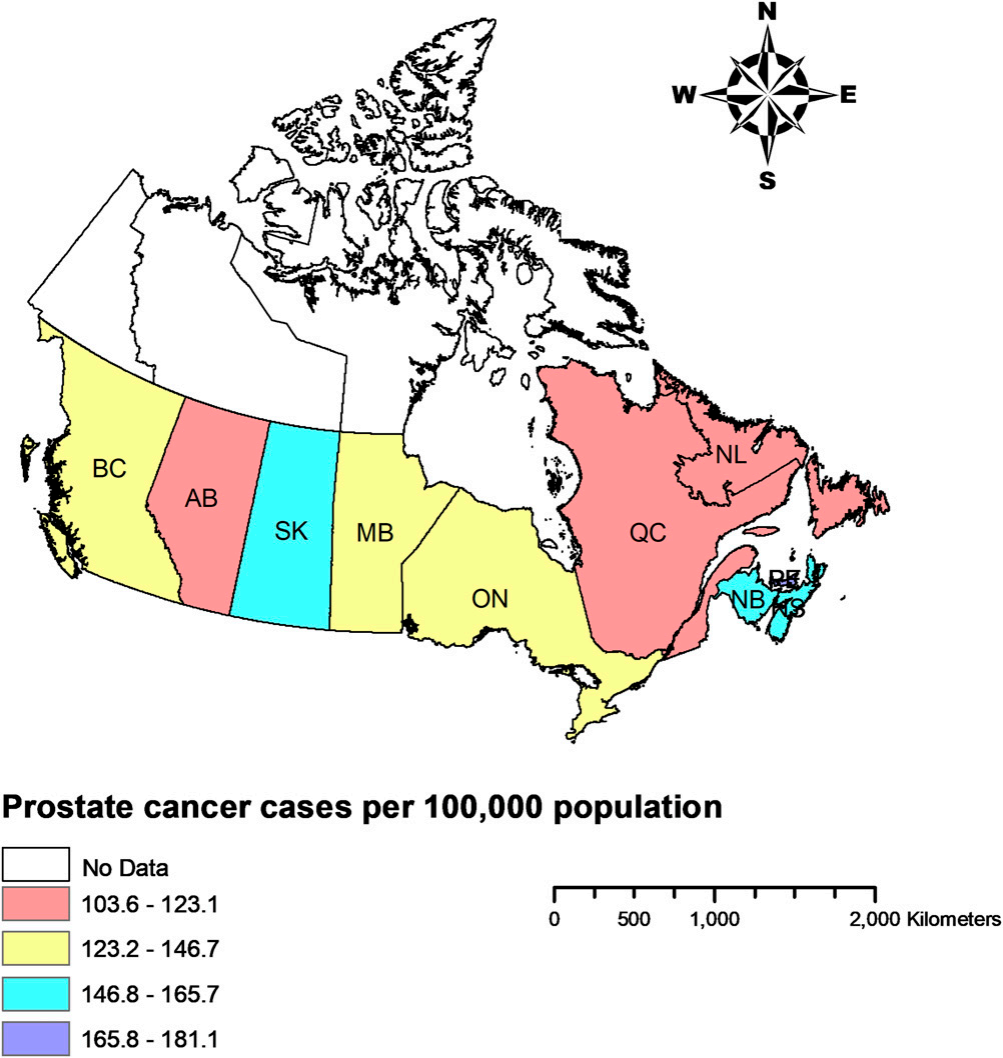
Average crude incidence rates of prostate cancer among males in Canadian provinces over the period 1992 to 2010 Note: British Columbia (BC), Alberta (AB), Saskatchewan (SK), Manitoba (MB), Ontario (ON), Quebec (QC), New Brunswick (NB), Nova Scotia (NS), Prince Edward Island (PE), Newfoundland and Labrador (NL).

Table 1 presents the national and provincial crude PCa incidence rates in Canada from 1992–2010. The crude incidence increased from 115 to 137 per 100 000 males in Canada, with a trend coefficient of 2.01 over the time period assessed (P < 0.000) but with a peak in 2007. This suggests that the incidence of new prostate cancer cases was rising amongst Canadian males although there was a decrease toward the end of the study time period for all provinces. The PCa incidence rate increased significantly in all provinces except in three of the four western provinces (British Columbia, Saskatchewan and Manitoba). The trend coefficient was negative for Manitoba, but not statistically significant in British Columbia and Saskatchewan. Table 1 reports and Figure 1 illustrates variation in the average crude PCa incidence rates across the Canadian provinces over the study period. As reported in Table 1 and shown in Figure 1, Prince Edward Island, New Brunswick, Saskatchewan and Nova Scotia had the highest average crude PCa incidence rates, whereas Quebec and Alberta had the lowest average crude PCa incidence rates.

**Table 1.** Crude incidence of prostate cancer per 100 000 among males in Canada and across its provinces from 1992 to 2010.

Year	BC	AB	SK	MB	ON	QC	NB	NS	PE	NL	Canada
1992	165.46	92.89	147.13	175.23	111.15	92.65	129.15	113.62	150.42	53.56	114.77
1993	192.92	112.34	175.10	192.18	119.62	111.47	174.56	141.17	174.17	85.70	132.31
1994	138.11	97.64	156.05	164.19	110.12	105.71	155.95	143.58	198.91	70.16	117.23
1995	118.42	96.52	132.28	145.74	100.92	88.09	122.53	109.40	145.36	68.31	103.02
1996	126.58	105.87	126.08	119.91	104.66	77.61	128.10	116.23	114.76	84.93	103.06
1997	135.02	101.76	128.15	129.13	119.23	79.36	146.20	135.60	168.31	86.77	111.47
1998	130.24	112.60	143.65	137.44	121.43	81.83	154.56	139.02	137.71	92.31	114.50
1999	150.53	114.76	164.20	127.10	118.92	85.66	147.52	151.68	138.34	108.40	118.26
2000	153.76	122.57	162.10	135.39	131.03	98.07	137.59	152.83	130.66	120.44	127.43
2001	151.88	157.20	181.04	145.52	144.78	105.56	151.78	173.52	215.20	148.55	140.66
2002	150.53	139.55	169.46	136.31	140.71	104.27	143.26	144.79	215.20	128.47	134.58
2003	136.80	148.37	175.78	128.94	136.01	117.10	144.68	158.58	215.20	104.38	135.00
2004	128.30	123.25	206.64	131.08	138.91	115.47	165.17	169.89	192.34	108.62	133.55
2005	133.69	119.26	183.21	109.53	152.27	115.74	183.68	169.89	169.26	118.87	137.99
2006	150.37	130.61	156.58	125.69	156.69	117.66	209.31	210.07	230.80	182.40	146.32
2007	168.33	125.09	190.67	134.67	160.35	119.31	219.28	205.48	253.89	184.45	151.41
2008	158.32	135.51	169.36	114.92	147.33	118.62	237.79	171.04	184.64	190.60	143.95
2009	151.48	123.49	146.66	127.59	142.04	118.64	203.07	164.94	218.00	203.66	138.60
2010	146.55	119.03	126.71	128.45	144.79	114.58	194.72	166.07	187.93	197.61	136.61
Average 1992–2010	146.70	119.91	160.04	137.32	131.63	103.55	165.73	154.60	181.11	123.06	128.46
Trend coefficients	0.03	1.88	1.10	−2.48	2.79	1.84	4.49	3.75	3.90	7.80	2.01
P-values	0.97	0.01	0.27	0.00	0.00	.00	.00	.00	.01	.00	.00

Note: British Columbia (BC), Alberta (AB), Saskatchewan (SK), Manitoba (MB), Ontario (ON), Quebec (QC), New Brunswick (NB), Nova Scotia (NS), Prince Edward Island (PE), Newfoundland and Labrador (NL).

#### Income and education inequalities in age-adjusted prostate cancer incidence

**Figure 2.**
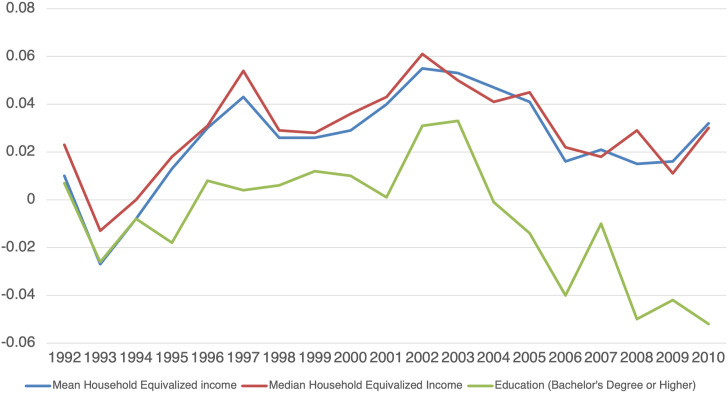
Trends in income and education inequalities in prostate cancer incidence among males in Canada: 1992 to 2010.

Table 2 and Figure 2 demonstrate the age-adjusted concentration indices of PCa incidence among Canadian males between 1992 and 2010 using income (both mean and median equivalized household income) and education as measures of SES. For both the mean and median income, the concentration indices were positive in all years except 1993 and 1994, indicating a higher incidence of PCa concentrated among males living in higher income CDs. About half of these positive values were statistically significant and most of the significant values were in the middle of the study time period (1996–2005). Linear trend analysis did not reveal any significant change in income inequalities in PCa over the study period.

**Table 2.** Income and education inequalities in prostate cancer incidence among males in Canada from 1992–2010.

Year	Age-Standardized C (95% confidence Interval)		
	Mean household equivalized Income	Median household equivalized income	Education (Bachelor's Degree or Higher)
1992	.01 (−.044 to .063)	.023 (−.025 to .071)	.007 (−.066 to .052)
1993	−.027 (−.076 to .021)	−.013 (−.061 to .035)	−.026 (−.089 to .037)
1994	−.008 (−.04 to .024)	0 (−.029 to .029)	−.008 (−.043 to .028)
1995	.013 (−.011 to .038)	.018 (−.006 to .041)	−.018 (−.049 to .013)
1996	.03 (.009 to .05)	.031 (.01 to .052)	.008 (−.020 to .035)
1997	.043 (.015 to .07)	.054 (.03 to .078)	.004 (−.030 to .039)
1998	.026 (-.002 to .055)	.029 (.004 to .055)	.006 (−.03 0to .042)
1999	.026 (−.002 to .054)	.028 (.004 to .052)	.012 (−.022 to .045)
2000	.029 (−.001 to .059)	.036 (.011 to .06)	.010 (−.028 to .048)
2001	.04 (.006 to .075)	.043 (.014 to .071)	.001 (−.036 to .038)
2002	.055 (.027 to .083)	.061 (.036 to .085)	.031 (.005 to .058)
2003	.053 (.023 to .082)	.05 (.025 to .075)	.033 (−.002 to .069)
2004	.047 (.025 to .069)	.041 (.017 to .064)	−.001 (−.025 to .022)
2005	.041 (.013 to .069)	.045 (.018 to .072)	−.014 (−.046 to .018)
2006	.016 (−.016 to .048)	.022 (−.008 to .052)	−.04 (-.073 to -.007)
2007	.021 (−.004 to .046)	.018 (−.004 to .041)	−.01 (-.038 to .017)
2008	.015 (−.019 to .049)	.029 (.002 to .057)	−.05 (-.082 to -.017)
2009	.016 (−.01 to .042)	.011 (−.012 to .034)	−.042 (-.072 to -.013)
2010	.032 (.006 to .058)	.03 (.007 to .054)	−.052 (-.087 to -.018)
Trend coefficient	.001	.0005	−.002
P-value	.183	.517	.030

**Note:** The inverse of the standard errors of the age-standardized C were applied as weights in the trend analyses.

The result of the age-adjusted concentration indices using education as an indicator of SES suggested that PCa incidence was significantly more concentrated among males living in lower education level CDs in recent years, that is, 2006–2010. The overall trend coefficient for age-adjusted concentration indices by education level was −0.0022 (*P* < 0.0300) demonstrating an increasing concentration of PCa incidence among males living in less-educated CDs over time.

## Discussion

The current study sought to quantify and examine trends in income and education-related inequalities in the incidence rate of PCa amongst Canadian males over time. Our descriptive results showed a growing number of new PCa cases in Canada between 1992 and 2010. This trend is consistent with globally increasing numbers of PCa incidence since the widespread uptake of PSA screening – particularly in developed countries.^6^

Globally, PCa incidence rates are highest in more developed parts of the world, including North America, Western and Northern Europe, and Australia.^4^ PCa incidence rates also increase with age.^40^ We reported crude incidence rates which show what is actually happening when there is no adjustment for age. Across the study time period, the crude incidence rates were generally highest in the three Maritime provinces of Prince Edward Island, New Brunswick and Nova Scotia. This was expected given that we reported crude rates and the age profile of these provinces are relatively older than the rest of the Canada.

Our study found that higher income was associated with an increased risk of developing PCa in many years especially in the middle of the study time period (1996–2005). In contrast, a lower level of education was associated with an increase in PCa incidence especially toward the end of the study time period (2006–2010). The increased incidence rate of PCa among populations of higher income in some years found in the current study was consistent with other studies showing a positive association between SES and higher PCa risk.^41,42^ The high incidence of PCa among high-income populations may be partially explained by increased opportunistic PSA screening among higher income males compared to males of lower income.^43,44^

Our study found lower levels of education to be associated with a higher risk of PCa among Canadian males in recent years. This is contrary to some studies that found higher levels of education to be a risk factor for PCa development^45^ and the contention that males who have attained higher levels of education are more likely to undergo PSA screening.^41,46,47^ Harsher work environments, work stress and shift work among males with lower education might partially explain our findings in that Canadian males with lower levels of education are at higher risk for PCa and if so may be a basis for primary prevention intervention. Also, the relative increase in PCa incidence over time might result from changes by education level in the prevalence of cigarette smoking or medical decisions over time.^33^ Targeting lower SES men for smoking prevention and prostate screening might be advised.^9^

This study is subject to some limitations. Firstly, we used area-based SES indicators and incidence to estimate socioeconomic inequalities. Although area-based SES indicators are commonly used to reflect populations of a given area, they do not necessarily reflect the individual characteristics accurately within that population. Since both area- and individual-based SES were shown to be independently correlated with health status,^48^ future work should assess the association between both area- and individual-based SES and PCa incidence in Canada. Secondly, as the CCP is administered every 5 years, we assigned the closest census years to each CCR studied, leaving the possibility that socioeconomic data may not have been as accurate as we had hoped. Thirdly, although the findings of our study provided insight into income and education inequalities in prostate cancer incidence over almost two decades in Canada, we could not assess changes in socioeconomic inequalities in prostate cancer incidence in recent years due to the data availability. Future work is required to update our study findings by using more current data when they become available.

## Conclusions

Overall, our study brings to light that, like other parts of the world, PCa is a public health concern in Canada. Higher use of opportunistic screening among high-income populations may explain the higher incidence rate among Canadian males of higher income. Nevertheless, this should be studied in further detail. Since we found a higher incidence rate of prostate cancer among less-educated males in Canada in recent years, risk-benefit investigation of primary prevention and opportunistic screening for less-educated males in Canada and beyond is advised.
